# Special

**Published:** 1888-03

**Authors:** 


// HALL'S
### JOURNAL OF HEALTH

TRUTH DEMANDS NO SACRIFICE; ERROR CAN MAKE NONE.

VOL. 35. MARCH, 1888 NO. 3.

### SPECIAL.

Owing to the unexpected absence in Europe of the Editors of *Earnest Words*, it has been deemed advisable to suspend its further publication for the present. In order, however, that those whose paid period of subscription has not expired, shall suffer no disappointment by this suspension, an arrangement has been made with HALL'S JOURNAL OF HEALTH to fill their several terms with issues of this journal, and we trust that this arrangement will prove satisfactory to all parties concerned.

We request our patrons in making remittances to us in sums under $5, to do so by postal orders or P. O. stamps.

